# Multimolecular Salivary Mucin Complex Is Altered in Saliva of Cigarette Smokers: Detection of Disulfide Bridges by Raman Spectroscopy

**DOI:** 10.1155/2013/168765

**Published:** 2012-12-26

**Authors:** Motoe Taniguchi, Junko Iizuka, Yukari Murata, Yumi Ito, Mariko Iwamiya, Hiroshi Mori, Yukio Hirata, Yoshiharu Mukai, Yuko Mikuni-Takagaki

**Affiliations:** ^1^Department of Functional Biology, Kanagawa Dental College, 82 Inaokacho, Yokosuka 238-8580, Japan; ^2^Department of Maxillofacial Diagnostic Science, Kanagawa Dental College, 82 Inaokacho, Yokosuka 238-8580, Japan; ^3^Department of Oral Medicine, Kanagawa Dental College, 82 Inaokacho, Yokosuka 238-8580, Japan; ^4^Department of Dental Sociology, Kanagawa Dental College, 82 Inaokacho, Yokosuka 238-8580, Japan; ^5^Yokohama Training Center, Kanagawa Dental College, 3-31-6 Turuyacho, Kanagawa-ku, Yokohama 221-0835, Japan; ^6^Department of Pathology, Tsurumi University School of Dental Medicine, Yokohama 230-8501, Japan; ^7^Clinical Laboratory, Kanagawa Dental College, 82 Inaokacho, Yokosuka 238-8580, Japan

## Abstract

Saliva contains mucins, which protect epithelial cells. We showed a smaller amount of salivary mucin, both MG1 and MG2, in the premenopausal female smokers than in their nonsmoking counterparts. Smokers' MG1, which contains almost 2% cysteine/half cystine in its amino acid residues, turned out to be chemically altered in the nonsmoker's saliva. The smaller acidic glycoprotein bands were detectable only in smoker's saliva in the range of 20–25 kDa and at 45 kDa, suggesting that degradation, at least in part, caused the reduction of MG1 mucin. This is in agreement with the previous finding that free radicals in cigarette smoke modify mucins in both sugar and protein moieties. Moreover, proteins such as amylase and albumin are bound to other proteins through disulfide bonds and are identifiable only after reduction with DTT. Confocal laser Raman microspectroscopy identified a disulfide stretch band of significantly stronger intensity per protein in the stimulated saliva of smokers alone. We conclude that the saliva of smokers, especially stimulated saliva, contains significantly more oxidized form of proteins with increased disulfide bridges, that reduces protection for oral epithelium. Raman microspectroscopy can be used for an easy detection of the damaged salivary proteins.

## 1. Introduction

Cigarette smoke contains free radicals, which can damage tissues [1, 2]. Saliva plays a role in the general defense system of the oral environment, and in addition to antioxidants, it contains immunoglobulins, antibacterial enzymes, and growth factors. Saliva also contains a mucous secretion to protect epithelial cells from mechanical as well as chemical challenges [3]. The secreted mucins MG1 and MG2 [4], which make large complexes with amylase, proline-rich proteins, statherin, histatin, and other proteins, form the first line of epithelial protection [5, 6]. Previous reports showed that free radicals degrade proteins [7, 8] and that mucins are modified in both sugar and protein moieties [9]. In addition, surface-exposed cysteine residues of proteins are particularly sensitive to oxidation by almost all forms of reactive oxygen species (ROS), and the oxidation of these sulfur-containing amino acid residues is reversible [10]. These proteins therefore serve as antioxidants [8]. In the airway of smokers, mucin expression/secretion is upregulated [11–14]. However, there is no test or assay by which to easily detect oxidized proteins in the saliva of smokers, and there is no good way to determine to what extent they are altered. We, therefore, collected protein components of saliva from both nonsmokers and smokers by immediately precipitating them with ethanol to separate them from low-molecular-weight sulfhydryl donors. We then examined actual disulfide bonds in the protein components in the saliva of smokers.

## 2. Material and Methods

### 2.1. Subjects and Populations, Collection and Storage of Saliva

Premenopausal females between 35 and 49 years of age were recruited after gaining the approval of the ethics committee of Kanagawa Dental College (number 10–04, 2010). We selected healthy volunteers with no significant medical history who were either nonsmokers, who had never smoked, or current smokers. The average ages of the 48 nonsmokers and the 10 smokers were 41.8 ± 3.9 and 40.0 ± 4.8 years, respectively. Subjects did not smoke for 3 hours after they ate lunch. Then whole saliva was collected by draining in a single session until 7.5 min had elapsed or until the volume reached 20 mL, whichever came first. Saliva was collected either under an unstimulated (resting) condition (R) or a stimulated condition by having subjects chew a 5-g piece of paraffin wax for 5 min immediately before collection (S). Saliva was maintained on ice and centrifuged within 1 hr of collection at 12,000 g for 30 min to remove cellular and other debris. The samples were immediately either subjected to 70% ethanol precipitation of proteins or to measurements for sulfhydryl residues.

### 2.2. Measurements of Sulfhydryl Residues

To estimate the concentration of sulfhydryl groups in saliva, the dithionitrobenzoic acid (DTNB) assay method was used as reported [15, 16] with L-cysteine as a standard. Fifty *μ*L of stock DTNB solution (10 mM in ethanol) was added to 1 mL of a solution containing 250 *μ*L of fresh saliva in a 0.25 mM Tris-HCl buffer at pH 8.3 in the presence or absence of L-ascorbic acid 2-phosphate (Asc2P) at 125 *μ*M. The sample was left at room temperature (22°C) to allow maximum color development, which was stable for at least 24 h. Absorption of the assay at 412 nm was determined before and after incubation, and the baseline values were subtracted. 

### 2.3. Protein Preparation, SDS-PAGE, and Staining

Seven volumes of ice-cold ethanol were mixed with 3 volumes of saliva, kept on ice for up to 1 hr, and centrifuged to collect the pellet, which was dried under vacuum, immediately weighed and dissolved in SDS sample buffer (0.3 M Tris-HCl buffer, pH 6.8, 6.25% SDS, 25% glycerol, and 0.1% bromophenol blue) containing 2 M urea at 10 mg/mL. Aliquots were heated at 94°C for 10 min in the presence or absence of 50 mM DTT. We purchased chemicals from Wako Pure Chemical Industries, Ltd., and SDS from BDH Chemicals Ltd. (London, UK). Five-*μ*L samples were electrophoresed on polyacrylamide gel with a gradient of either 5–20% or 10–20% and with Precision Plus Protein Standard Kaleidoscope (Bio-Rad Laboratories, Hercules, CA) as a standard. Gels were fixed (50% methanol and 10% acetic acid), stained with Stains-All (Bio-Rad Laboratories, Ltd.) [17], washed overnight with two changes of 25% 2-propanol, and rinsed with two changes of H_2_O. After recording scanned images, gels were treated with fixative again to remove the dye and restained for protein with Coomassie Brilliant Blue-R250 (Sigma Japan, Ltd., Tokyo, Japan). We selected four samples at random from each group (Figures 3 and 4). 

### 2.4. Western Blot Analysis

After the SDS-PAGE, proteins were electrotransferred and incubated for 1 h with primary antibodies [18]. Goat polyclonal antibody for human serum albumin at 1 : 10,000 (ab19183, Abcam PLC, Cambridge, UK), rabbit monoclonal antibody for human *α*-amylase at 1 : 24,000 (3796S, Cell Signaling Technology, Inc., Danvers, MA), and mouse polyclonal antibody for human MG1 mucin (MUC5B gene product) at 1 : 500 (H00727897-A01, Abnova Corporation, Taipei, Taiwan) were used. Images recorded with LAS-3000 (Fuji Photo Film Co. Ltd., Kaisei-Machi, Japan) were digitized with ImageJ software (http://rsb.info.nih.gov/ij/).

### 2.5. Detection of Disulfide Bonds in Saliva Protein by Confocal Laser Raman Microspectroscopy

A portion of the wet pellet described in Section 2\tmspace +\thinmuskip {.1667em}2.3 above was placed between two glass slides before drying as a thin film in a container filled with N_2_ gas. A Nicolet Almega XR Dispersive Raman microscope system (not a transmission type as FT-IR) equipped with the OMNIC Atl*μ*s imaging software program (Thermo Fisher Scientific, Inc., MA) and a high-brightness, low-intensity laser operating at 780 nm was used. Intensities of the amide I peak at 1740–1550 cm^−1^ [19], the S-S stretch at 600–470 cm^−1^ [20], and SH at 2,480–2,620 cm^−1^ [21] were measured.

### 2.6. Statistical Analysis

To test the statistical significance of all measurements, we used Fisher's exact probability test for the smokers and for the nonsmokers. We judged a difference to be statistically significant when *P* < 0.05.

## 3. Results

### 3.1. Sulfhydryl Content of Salivary Proteins

**Figure 1:**
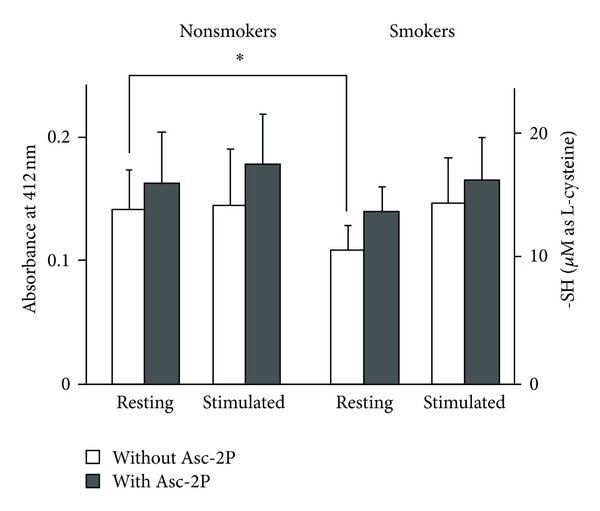
Sulfhydryl residues in the saliva of smokers and nonsmokers collected under stimulated and unstimulated (resting) conditions were compared separately with or without Asc-2P. Asterisks represent significant differences (*P* < 0.05).

The DTNB assay showed that the content of sulfhydryl residues in the saliva of nonsmokers and stimulated saliva (S) was greater than that in the saliva of smokers and unstimulated saliva (R), respectively (Figure 1). Among untreated saliva, smokers' unstimulated (resting) saliva gave significantly lower values than that of nonsmokers. The increments by reduction with Asc2P were not significantly different from each other (data not shown). 

**Figure 2:**
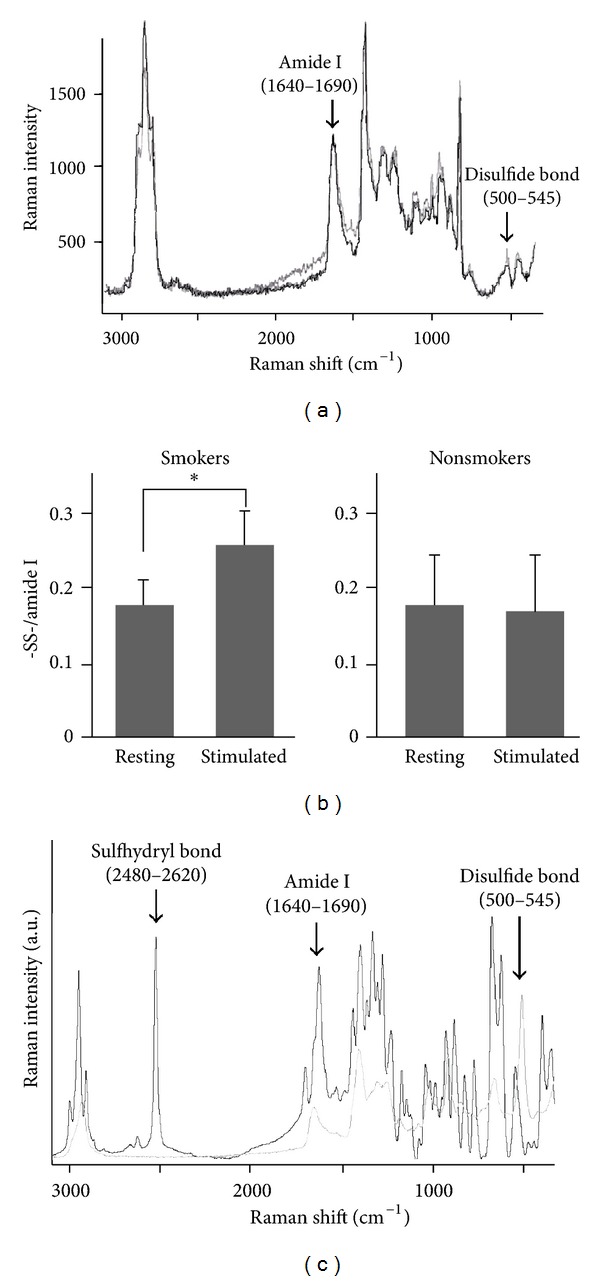
Raman spectra of S-S stretch and amide I derived from disulfide bonds of saliva proteins (a) and the areal ratio of disulfide residues per amide I (b). The black line and grey line correspond to stimulated saliva and unstimulated (resting)saliva, respectively. (c) Spectra similar to that of (a) of crystalline glutathione, GSH and GSSG forms, are presented with black and grey lines, respectively.

From the Raman profiles (Figure 2(a)), provided the intensity of the S-S stretch bands divided by the corresponding amide I, -SS-/protein ratio (Figure 2(b)), which was significantly higher in the smokers' stimulated saliva smokers' saliva than in all others (at *P* < 0.005 by *t*-test). The spectrum of glutathione crystals showed in Figure 2(c) a conspicuous peak at around 2,500 cm^−1^ for GSH and at 510 cm^−1^ for GSSG. If such GSH is weighed and dissolved in water in the absence of Asc2P, about 90% of the expected absorbance at 412 nm was attained by the DTNB method. By leaving the GSH crystals in the air, both the 2,500-cm^−1^ peak in the Raman spectrum and the 412-nm absorbance were significantly reduced. Peaks at 2,500 cm^−1^ and 510 cm^−1^ can be used as a measure of oxidation. 

### 3.2. Identification of Oxidized Salivary Proteins 

**Figure 3:**
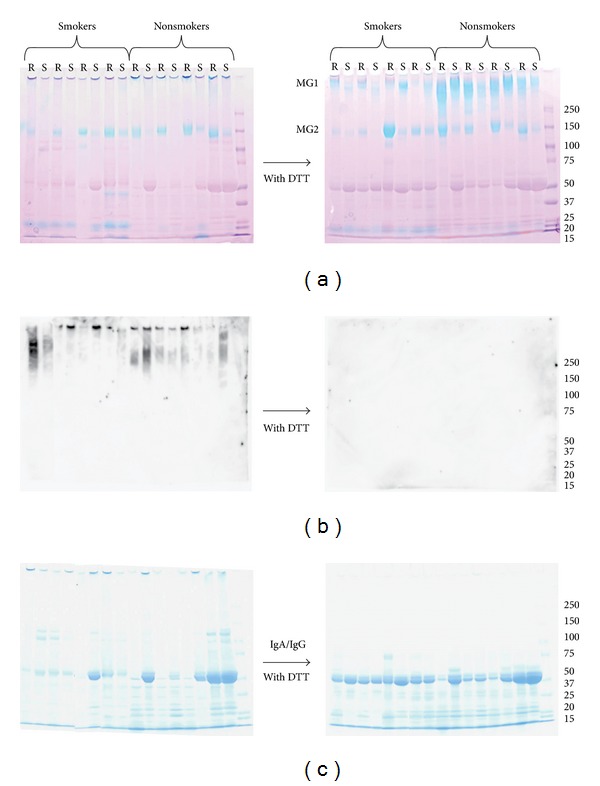
SDS-PAGE profiles of saliva proteins from smokers and nonsmokers on a 4–20% gradient gel visualized by Stains-All staining (a), Western blot with MUC5B antibody (b), and CBB protein staining (c). Portions from the same samples were treated with (right panels) and without (left panels) reducing agent DTT. Sizes of standard molecules run in the last lane are shown to the right of the gel. Saliva samples collected under unstimulated (resting) and stimulated conditions are shown in lanes R and S, respectively.

All the samples were analyzed by SDS-PAGE either with or without reducing agent.  Figure 3 shows the results of 4 smokers and 4 nonsmokers. Among the blue bands of acidic glycoproteins stained with Stains-All (Figure 3(a)), the MG2 band appeared around 150 kDa regardless of reduction or smoking background. On the other hand, the MG1 band of nonsmokers was apparent above 250 kDa after reduction with DTT. The MG1 band of smokers both in (S) and (R) saliva was much less significant. Instead, an alternative smaller blue band of about 20 to 25 kDa was distinct only in saliva of smokers. In samples of some smokers, staining of another distinct blue band was intense around 45 kDa. A common feature of the two major salivary mucins, MG1 and MG2, is that the intensity of mucin bands derived from the unstimulated saliva is almost always much higher than that from stimulated saliva. This also holds true with regard to the blue staining of the nonreduced samples, too large to be included in the gel. Commercially available MUC5B antibody detected MG1 of smaller size only without DTT (Figure 3(b)). Blue-staining MG1 with sulfate residues was not detectable. CBB staining, on the other hand, showed another distinct protein band of ~50 kDa (Figure 3(c)).

**Figure 4:**
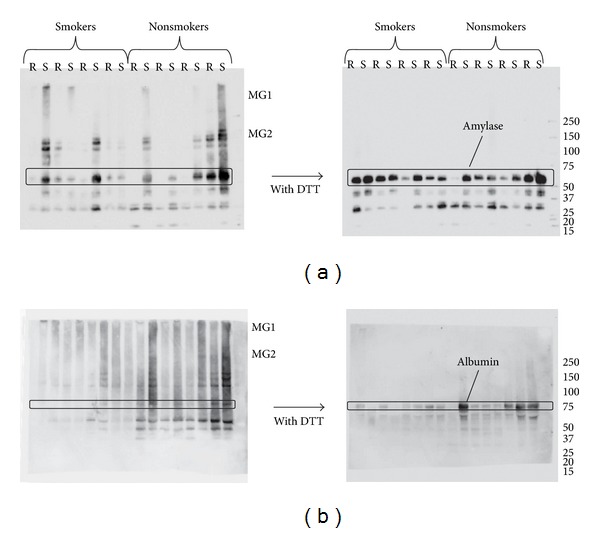
Western blot profiles detected with antibodies against (a) salivary amylase (top panels) and (b) serum albumin (bottom panels). Samples were treated either with (right panels) or without (left panels) reducing agent DTT.

While the intensity of the protein band of nonsmokers was comparable with and without DTT, it increased by reduction of the saliva samples of smokers. In general, more proteins, that is, stronger bands, were found in stimulated saliva, a result opposite to that of mucins. To further confirm the aforementioned results of potential oxidation and binding to other proteins in saliva, two major salivary proteins, albumin and amylase, were characterized by Western blotting with and without DTT reduction (Figure 4). In Figure 4(a), amylase antibody is bound to the 50 kDa band, which is seen in Figures 3(a) and 3(c) together with additional bands. Without reduction, antigenic reaction was seen in the area where immunoglobulins appeared. While the staining intensity of amylase bands in a few samples showed no increase by reduction with DTT, increased staining was seen in most samples. The increase in smokers was more significant than that of nonsmokers. There was no major difference, however, between the results of smokers and nonsmokers. On the other hand, specific albumin staining after reduction was distinct from that without reduction (Figure 4(b)). Without reduction, the albumin band around 67 kDa was not apparent. Instead, intense smears including the areas of MG1 were found. Also, the overall staining was stronger in the samples from nonsmokers regardless of reduction. Therefore, we compared the increments of the specific albumin band intensity by the inclusion of DTT to that of the band after reduction (see Figure 4(b), right panel). The intensities are 0.73 ± 0.21 for smokers and 0.48 ± 0.35 for nonsmokers. The *P* value of 0.038 showed that the difference was significant.

## 4. Discussion and Conclusions

Although saliva contains antioxidant defense systems to counteract the toxic effect of radical species formed by superoxide dismutase (SOD), glutathione peroxidase (GSH-Px), and other enzymes, cigarette smoke contains oxidants of other types as well, including oxygen-free radicals and volatile aldehydes, which damage biomolecules [22–25]. Mucins are susceptible to attack by reactive oxygen species during which terminal sugars are lost and both protein and sugar moieties are fragmented [9]. We studied components of saliva including mucins and showed that there is less multimolecular mucin complex in the saliva of smokers. Although blue staining of MG1 band decreases, the levels of MG2 and MUC5B antibody-reacting materials under nonreducing conditions are not that different between smokers and nonsmokers (Figures 3(a) and 3(b)). Therefore, acidic residues such as sulfated sugars may be specifically decreased in smokers' MG1 by degradation resulting in much less MG1 in saliva of smokers, especially in stimulated saliva. Lower MG1/MG2 levels in the stimulated saliva samples can be explained by increased contributions of parotid saliva, which is prevalent under that condition. Although we lack information regarding saliva flow rate, no significant correlations were previously found between mucin levels of stimulated saliva and age or flow rate [4]. Of note, a significant amount of smaller acidic glycoprotein bands appeared only in saliva of smokers. Also mainly in smokers' saliva, CBB staining of a distinct protein band of 50 kDa, which turned out to be amylase, was intensified together with several other protein bands after reduction with DTT. In addition, the albumin band detected by antibody binding showed not only that the albumin content decreased significantly in the saliva of smokers but also that the ratio of albumin bound to other proteins including MG1 by the disulfide bridges is significantly higher in the smokers' saliva. By using confocal laser Raman microspectroscopy, we confirmed the increased S-S stretch band derived from protein disulfide bonds in the stimulated saliva (S) of smokers. While significantly altered multimolecular mucin complex was reported in stimulated saliva previously [5, 6], we detected more disulfide residues as well as a higher rate of released proteins upon reduction by DTT in the samples of smokers' saliva than in those of nonsmokers'. MG2 mucin contains no cysteine residues, and we did not distinguish any difference between the characteristics of MG2 in the saliva of smokers and that of nonsmokers. It was also true with immunoglobulins. MG1 with the abundant cysteine residues may function as the surface-exposed reactive cysteine, which was previously reported [8, 10]. Our result agrees with that of Levine et al. who reported that DTT increases binding of MG1 alone to 1-anilino-8-naphthalene sulfonate [26]. Importantly, Brock et al. reported that there is a significant decrease of GSH-Px activity in male smokers compared to nonsmokers (*P* < 0.05) resulting in an oxidant/antioxidant imbalance [24]. The reason why such an apparent increase in the extent of protein oxidation was detectable in smokers' saliva in our study, we believe, is that we generally treated each saliva sample immediately after collection so that the chemical and enzymatic modifications are minimal. Complex formation with statherin and proline-rich proteins, PRPs, which do not contain cysteine, reported by Iontcheva et al. [6] is yet to be determined. In our future studies, we will address the actual degradation and oxidation mechanisms of salivary mucin MG1 by the radical species in the saliva of female smokers.
